# Treatment‐Limiting Decisions in Neurointensive Care: Withholding or Withdrawal of Life‐Sustaining Measures

**DOI:** 10.1111/aas.70202

**Published:** 2026-02-22

**Authors:** Isabella Carvalho Ankerstjerne, Anne‐Sophie Worm Fenger, Markus Harboe Olsen, Ove Bergdal, Daniel Kondziella, Helene Ravnholt Jensen, Gorm Greisen, Tiit Illimar Mathiesen, Kirsten Møller

**Affiliations:** ^1^ Department of Neuroanaesthesiology The Neuroscience Centre, Copenhagen University Hospital – Rigshospitalet Copenhagen Denmark; ^2^ Department of Neurosurgery The Neuroscience Centre, Copenhagen University Hospital – Rigshospitalet Copenhagen Denmark; ^3^ Department of Neurology The Neuroscience Centre, Copenhagen University Hospital – Rigshospitalet Copenhagen Denmark; ^4^ Department of Clinical Medicine Faculty of Health and Medical Sciences, University of Copenhagen Copenhagen Denmark; ^5^ Department of Neonatology The Neuroscience Centre, Copenhagen University Hospital – Rigshospitalet Copenhagen Denmark; ^6^ Department of Clinical Neuroscience Karolinska Instituttet Stockholm Sweden

**Keywords:** acute brain injury, ethics, prognosis, spinal cord injury, treatment‐limiting decision

## Abstract

**Background:**

Treatment‐limiting decisions (TLDs) in neurointensive care are frequently reported with no distinction between withholding and withdrawal of treatment. We investigated the proportion of patients subjected to withholding and withdrawal of life‐sustaining treatment during neurointensive care, and the association with mortality.

**Methods:**

This retrospective observational cohort study included all adult (≥ 18 years) neurocritically ill patients admitted to the neurointensive care unit (neuro‐ICU), Rigshospitalet, Denmark, for a primary CNS injury during the period from July 2019 to February 2022. Patients were categorised into the following three groups: full treatment, withholding, or withdrawal of life‐sustaining treatment. We compared 30‐day all‐cause mortality of the three groups by a Cox proportional‐hazards model and by calculating the mean restricted survival time at 30 days.

**Results:**

Of 694 eligible patients, a decision to withhold or withdraw treatment was made for 50 (7.2%) and 124 (17.9%) patients, respectively. Patients subjected to withholding were older and had a higher Charlson Comorbidity Index, whereas those subjected to withdrawal had a lower Glasgow Coma Score (GCS) and a higher Acute Physiology and Chronic Health Evaluation II score. While the primary neurological injury was the main reason for the treatment‐limiting decision, and comorbidity contributed in both groups, a history of cardiac arrest was also stated as a reason for withdrawing, and non‐neurological injury as a reason for withholding therapy. The 30‐day case fatality ratio was 32%, 98% and 8.1% for patients subjected to withholding, withdrawal and full treatment, respectively, corresponding to a mean restricted survival time at 30 days of 24 (95% CI, 21–27), 10.0 (8.2–12) and 29 (28, 29) days.

**Conclusion:**

In this study, TLDs were made in one out of four neuro‐ICU patients. Furthermore, neurocritically ill patients subjected to withholding treatment had markedly higher survival than those subjected to withdrawal.

**Editorial Comment:**

In this assessment of treatment‐limiting decisions in neuro‐intensive care cases, the authors present how withholding escalating ICU treatment can occur in case conditions that differ from those where active ICU treatment is withdrawn.

## 1 Introduction

Treatment in the neurointensive care unit (neuro‐ICU) focuses on minimising the consequences of the primary neurological injury and preventing secondary injury [1, 2]. Regardless of the underlying condition, the prognosis is often uncertain early during admission [1, 3, 4], mandating full active treatment. However, later during the clinical course, reconsideration of the level of treatment may be appropriate [1, 3]. Accordingly, treatment‐limiting decisions (TLDs) may be made when initiation or continuation of aggressive ICU care is considered futile or if potential outcomes are considered undesirable. Futility denotes a situation when treatment no longer affects prognosis, while prioritisation and assessment of desirability involve explicit or hidden value judgements. Moreover, TLDs may be required if care is delivered under conditions when resources are limited, such as mass casualties or a pandemic [5]. Finally, decision‐making involves prognostication with inherent risk of mistakes. Commonly, TLDs are supported by an implicit consensus that the patient's chance of recovering to an acceptable quality of life is slim, and further active treatment for the purpose of survival is either unlikely to improve that chance or ‘associated with disproportionate suffering’ [3].

In a Danish context, the Health Act provides the legal framework for the treatment of patients in both the private and the public health sector. Full active treatment represents the default; a patient who is capable of decision‐making must provide informed consent to the treatment and its risks, whereas a patient who is temporarily incapable of decision‐making may be treated actively by an emergency clause. However, the treating physician has the right not to provide treatment that they deem to be irrelevant, unjustified, or futile for that specific patient. Conversely, a patient who is capable of decision‐making may engage in advanced care planning to refrain from active treatment in the case of life‐threatening or debilitating illness.

TLDs can be divided into withholding or withdrawal of life‐sustaining measures and are usually determined by the severity of the injury or disease, as well as the patient's comorbidities and current condition [1, 6]. Treatment is typically withheld when prognosis is considered unfavourable, regardless of intensity of treatment, while withdrawal is motivated when patients fail to improve or stabilise despite aggressive care. Importantly, prognostic uncertainty is inevitable, which makes TLDs difficult [1, 4]. In some cases, aggressive treatment will be pursued, while in other cases, a possible unfavourable functional outcome will lead to an early TLD [3, 7, 8]. In Europe, TLDs were reported in 6%–12% of ICU‐treated patients, but previous studies failed to differentiate treatment withdrawal from withholding treatment [9, 10, 11]. TLDs are part of routine intensive care, and it is essential to realise their fundamental ethical connotations.

The aim of this retrospective study was to investigate the use of TLDs and the reasons for making TLDs in a cohort of patients admitted to the neuro‐ICU. We recorded the proportion of patients who had life‐sustaining measures either withheld or withdrawn and compared the characteristics of the underlying reason for TLDs, the specific treatment limitations and 30‐day mortality between groups.

By recording withholding and withdrawal of treatment separately, we aimed to investigate the association of these practices with patient characteristics and outcomes. We expected similarly bad outcomes regardless of withholding or withdrawal, since both practices would reflect an assessment of the futility of further care.

## 2 Methods

This retrospective, observational cohort study of patients admitted to the neuro‐ICU of Rigshospitalet, Denmark, was approved by the Centre for Regional Development in the Capital Region of Denmark (R‐21060570, 03 November 2021) and the Danish Data Protection Agency (P‐2021‐760, 12 November 2021). According to Danish law, no consent was needed from patients or the next of kin.

### 2.1 Study Population

We screened all patients admitted to the neuro‐ICU at Rigshospitalet, Denmark, from July 2019 to February 2022 with traumatic brain injury (TBI), subarachnoid haemorrhage (SAH), intracerebral haemorrhage (ICH), spinal cord injury (SCI), intracranial tumours, and other neurological or neurosurgical conditions requiring neurointensive care. Inclusion criteria were age ≥ 18 years and length of stay in the neuro‐ICU > 24 h. Exclusion criteria were admission for COVID‐19 (as some patients were admitted to the neuro‐ICU due to capacity problems in other ICUs rather than due to a primary neurological condition), another non‐CNS‐related condition as the primary diagnosis, or admission solely for the purpose of organ donation or diagnostic workup prior to organ donation.

### 2.2 Data Collection

Data were collected from electronic patient charts (Sundhedsplatformen, Epic/NNIT, Verona, WI 53593, USA) and included age, sex, primary admission diagnosis and the first recorded Glasgow Coma Score (GCS) at the time of injury or disease onset (ictus), time from ictus to admission to a trauma centre or emergency room, duration of mechanical ventilation and 30‐day mortality from date of admission to the neuro‐ICU. Charlson Comorbidity Index was recorded for each patient according to registered diagnoses of chronic disease, and Acute Physiology and Chronic Health Evaluation (APACHE) II score was calculated at admission to the neuro‐ICU as a measurement of disease severity.

All data were collected and manually entered into REDCap (Research Electronic Data Capture Software, Vanderbilt University, USA) by the first author (ICA), based on thorough review of each patient's chart. Data collection procedures, variable definitions and extraction protocols were predefined and continuously aligned with the study supervisors and co‐authors to ensure consistency and validity. Structured data (e.g., laboratory values) were exported directly when available. All data handling was carried out in accordance with the European Union General Data Protection Regulation.

### 2.3 Treatment‐Limiting Decision

The level of treatment is recorded as a separate entry in the field, ‘Level of treatment’ in the electronic patient chart, which is immediately visible once the patient's file is opened. It is required to be accompanied by an explanatory case note, in which the reasons for the TLD are made and the restricted treatments can be described.

**FIGURE 1:**
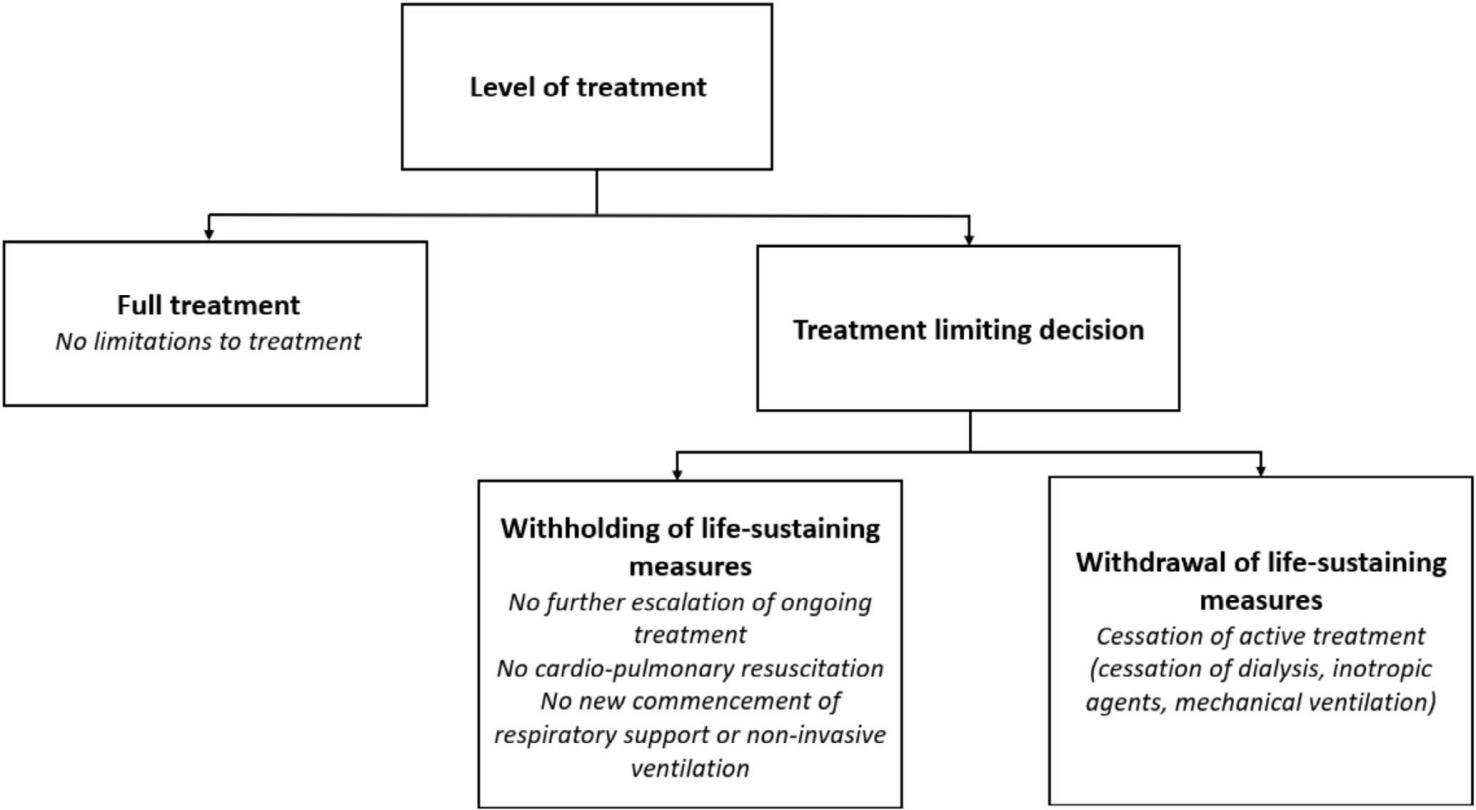
Level of treatment.

We defined the level of treatment as full treatment if no restriction on the patient's treatment had been recorded in the ‘Level of treatment’ field (with or without an explanatory case note). If a TLD had been recorded, we searched the case notes to establish the time at which it was made, as well as whether it could be characterised as withholding or withdrawal of life‐sustaining measures. Withholding of life‐sustaining measures (henceforth referred to as ‘withholding’) was defined as ‘no further escalation of ongoing treatment’, ‘no attempt of resuscitation in the case of cardiac arrest’ and/or ‘no new commencement of respiratory support or non‐invasive ventilation’ (Figure 1). Withdrawal of life‐sustaining measures (henceforth referred to as ‘withdrawal’) was defined as cessation of active treatment (e.g., dialysis, vasoactive medication and/or mechanical ventilation). Only the most extensive decision was recorded; thus, if a patient at any time during admission to the neuro‐ICU underwent both withholding and withdrawal of life‐sustaining measures at different times, the decision to withdraw would be recorded, rather than the decision to withhold. Case notes were searched for a possible change of a TLD, that is if the patient had been subjected to withholding, and full treatment was reintroduced. We further recorded the time from hospitalisation to the TLD and the specified primary and secondary reasons for the TLD. The primary reason for a TLD was recorded as either neurological, and due to either primary or secondary neurological injury, unknown or non‐neurological. Patients who became organ donors during admission were registered as subject to withdrawal. Hence, all patients were allocated to one of the following three groups: (1) full active treatment, (2) withholding of life‐sustaining measures, and (3) withdrawal of life‐sustaining measures.

### 2.4 Statistical Analysis

Statistical analyses were carried out using R (R Core Team, Vienna, Austria). Descriptive continuous data were not normally distributed and were presented using median and interquartile ranges (IQR), while categorical data were presented as numbers and percentages.

Normality of continuous variables was assessed visually using histograms and Q‐Q plots. If the variables were not normally distributed, we applied non‐parametric methods for all comparisons of continuous data.

Age, GCS, APACHE II and Charlson Comorbidity Index were compared between groups using the Wilcoxon signed rank test. 30‐day survival from admission to the neuro‐ICU between the three groups was presented in a Kaplan‐Meier plot and analysed using a Cox proportional hazards model investigating the three treatment level groups with Charlson Comorbidity Index as a covariate. Assumptions for the Cox proportional hazards model were tested, and the results were presented with hazard ratio (HR) and 95% confidence intervals (CI). Similarly, these survival analyses were also carried out by comparing 30‐day survival between patients subjected to withholding and withdrawal, respectively, with the time of the TLD as the starting point. Finally, we carried out secondary analyses of 30‐day restricted mean survival time comparing full treatment with either withholding or withdrawal from admission to the neuro‐ICU and between withholding and withdrawal. All analyses were explorative and hypothesis‐generating. Therefore, we did not apply any correction for multiple testing, and the alpha level was set at 0.05 throughout. Finally, Venn diagrams were created to visualise the type of TLDs for each group.

## 3 Results

**FIGURE 2:**
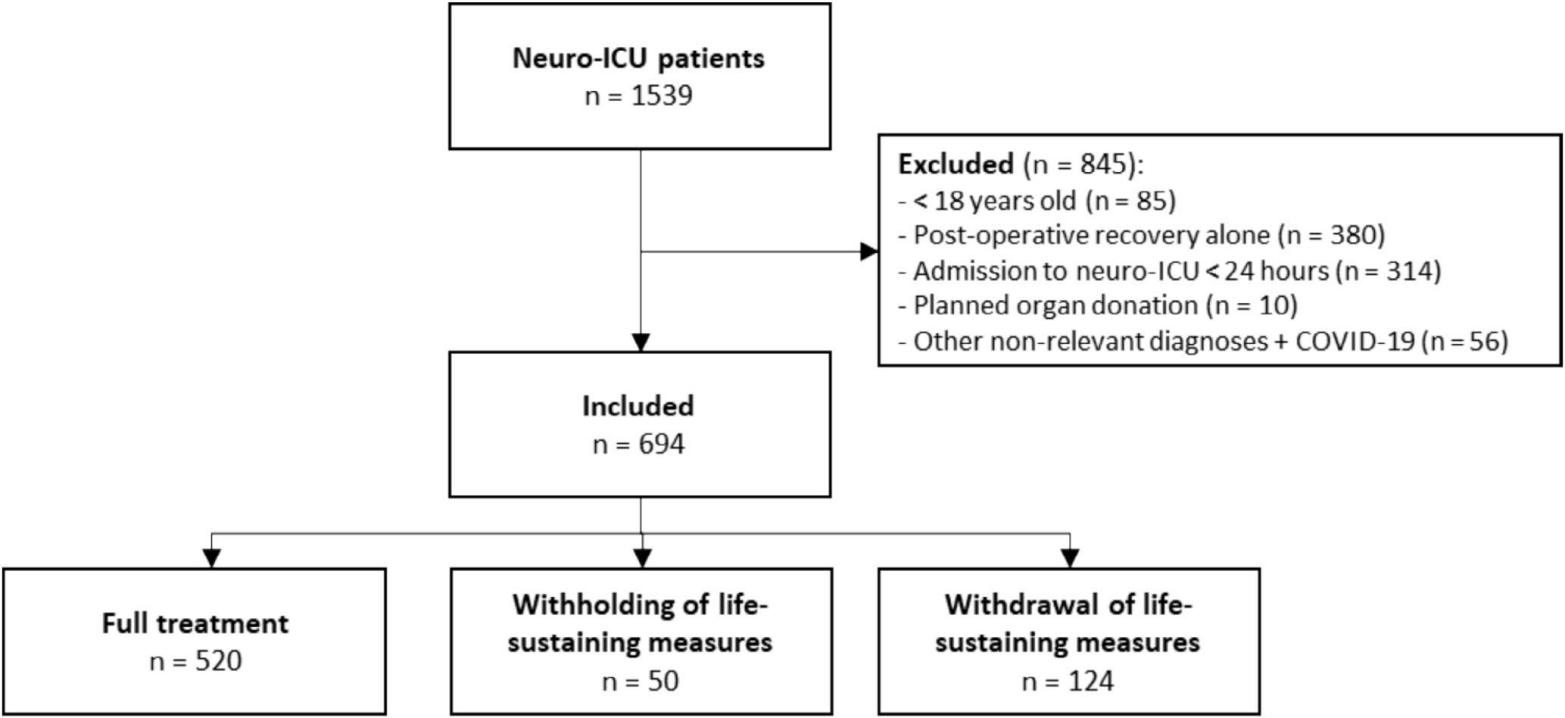
Flowchart for identifying patients.

We screened 1539 patients for eligibility, of whom 694 patients were included (Figure 2). Of those, 174 patients (25%) received at least one TLD during admission. Patients subjected to withholding (*n* = 50) were older and had more comorbidities than patients in the groups subjected to full treatment (*n* = 520) and withdrawal (*n* = 124), respectively (Age: *p* = 0.001, *p* < 0.05; Charlson Comorbidity Index: *p* < 0.001, *p* < 0.01) (Table 1, Supporting Information). Conversely, patients subjected to withdrawal (*n* = 124) had a lower initial GCS and a higher APACHE II score than patients in the groups subjected to full treatment and withholding, respectively (GCS: *p* < 0.001 for both comparisons; APACHE II: *p* < 0.001 for both comparisons) (Table 1, Supporting Information). The primary neurological injury was the most prevalent reason for a TLD in both groups, but more so in the withdrawal group (Table 2).

**TABLE 1:** Demographic data and patient characteristics.

	Full treatment	Withholding of life‐sustaining measures	Withdrawal of life‐sustaining measures
*n*	520	50	124
Age (years)	56 [46–66]	66 [48–75]	60 [52–67]
Sex = male	280 (54)	24 (48)	72 (58)
Charlson comorbidity index (total points)	2 [1–4]	4 [3–6]	3 [2–4]
Diagnosis			
aSAH	75 (14)	6 (12)	19 (15)
sICH	129 (25)	18 (36)	41 (33)
TBI	78 (15)	5 (10)	12 (10)
Other (cerebrovascular/tumour/SCI)^a^	151 (29)	15 (30)	29 (23)
Other (neurological/neuroinfection/neuroinflammation)^b^	87 (17)	6 (12)	23 (19)
First GCS (total score)	14 [9–15]	12 [7–15]	6 [3–13]
APACHE II (points)	18 [13–23]	20 [16–24]	23 [20–26]
Mechanical ventilation	278 (54)	33 (66)	107 (86)
Duration			
Ictus to hospital admission (h)	2 [0.8–15.6]	1.9 [1–16.7]	1.2 [0.7–4.5]
Hospital admission to neuro‐ICU (h)	3.3 [0.5–17.7]	2.8 [0.7–31.7]	1.5 [0.2–6.1]
Length of stay, intensive care unit (days)	3.9 [1.5–10.9]	9.1 [3.2–17.7]	2.2 [1.3–4.9]
30‐day mortality	42 (8)	16 (32)	121 (98)

*Note:* Data are median [IQR] or number (%).

Abbreviations: APACHE II, Acute Physiology and Chronic Health Evaluation II; aSAH, aneurysmal subarachnoid haemorrhage; ER/TC, emergency room/trauma centre; GCS, Glasgow Coma Scale; *n*, number; sICH, spontaneous intracerebral haemorrhage; TBI, traumatic brain injury.

^a^ Other (cerebrovascular/tumour/spinal cord injury (SCI)): ICH secondary to other primary lesion, arteriovenous malformation, ischaemic stroke, non‐aneurismal SAH, intracerebral tumour, spinal cord injury.

^b^ Other (neurological/neuroinfection/neuroinflammation): hypoxic/anoxic brain injury, Guillain‐Barré syndrome, encephalitis, myasthenia gravis, neuroinfection, neuroinflammation, status epilepticus.

**TABLE 2:** Reasons for treatment‐limiting decisions.

	Withholding of life‐sustaining measures	Withdrawal of life‐sustaining measures
Total number	50	124
Time from neuro‐ICU admission to TLD (hours)	28.2 [15.2, 218.3]	35.7 [15.1, 98.8]
Main reason for TLD (%)		
Neurological injury (primary)	34 (72)	119 (96)
Neurological injury (secondary)	0 (0)	1 (1)
Non‐neurological injury	8 (17)	3 (2)
Unknown reason	8 (16)	1 (1)
Additional reason for TLD (%)		
Age	1 (2)	1 (1)
Comorbidities	23 (46)	27 (22)
Cardiac arrest in conjunction with injury or disease onset	2 (4)	20 (16)

*Note:* Data are median [IQR] or number (%).

Abbreviation: TLD, treatment‐limiting decision.

**FIGURE 3:**
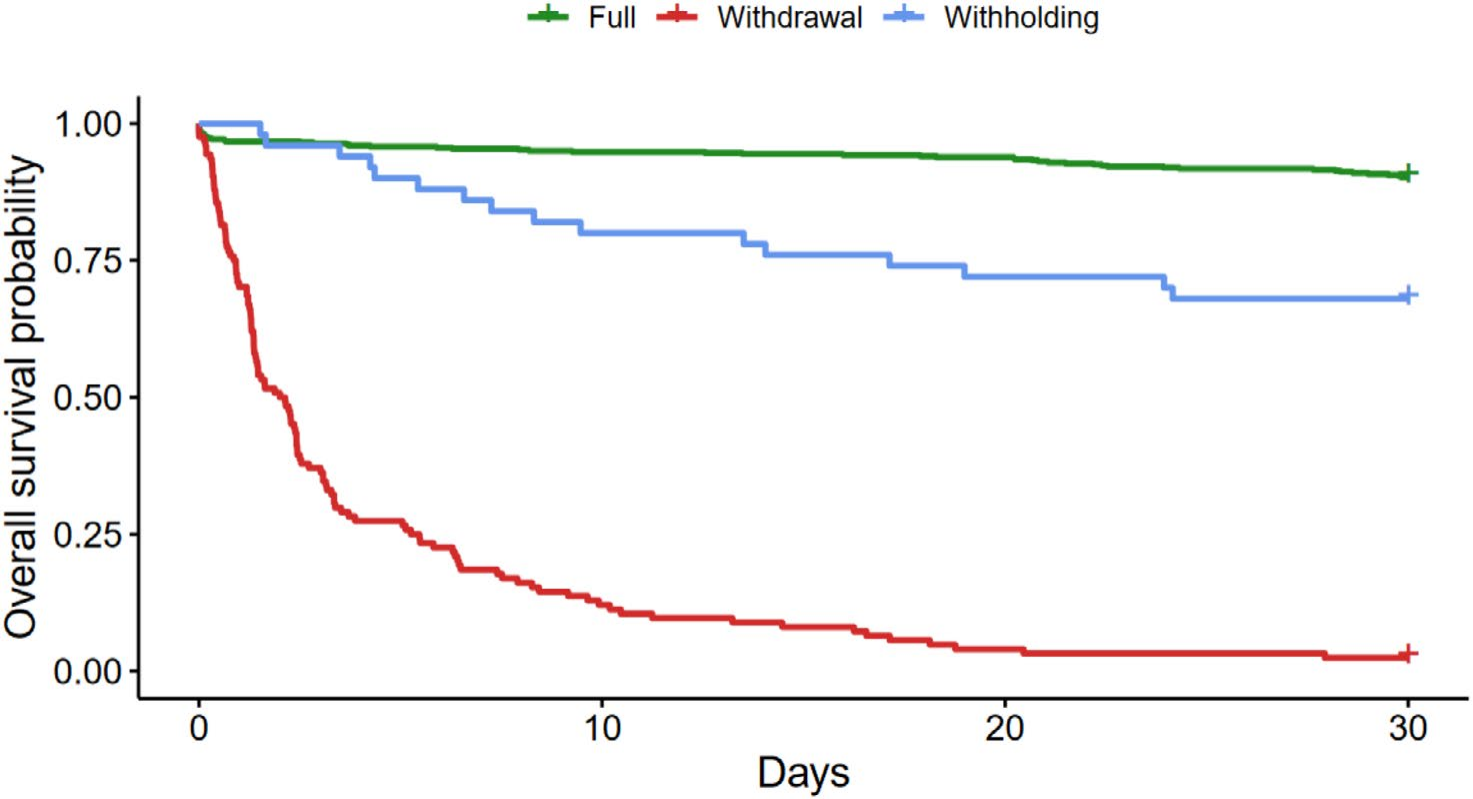
30‐day mortality after admission to the neuro‐ICU (Kaplan‐Meier plot).

**FIGURE 4:**
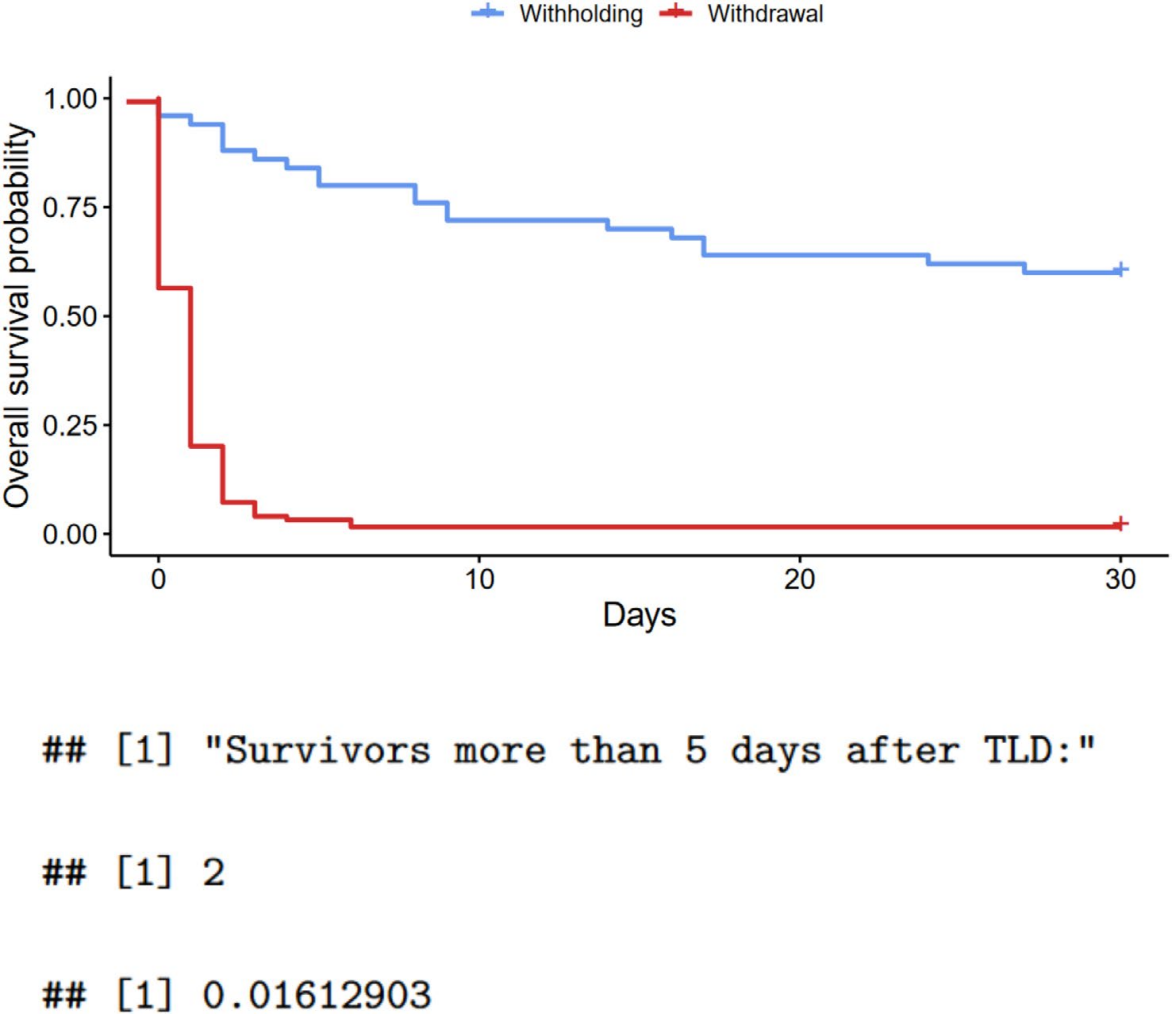
30‐day mortality after a treatment‐limiting decision (Kaplan‐Meier plot).

In the group subjected to the decision to withdraw treatment, 97.6% had died within 30 days after admission, compared to 32% in those subjected to withholding and 8.1% receiving full treatment (Figure 3). This corresponded to a restricted mean survival time at 30 days of 10.0 (95% CI 8.2–11.7, *p* < 0.001 compared to full treatment and compared to withholding) days for the withdrawal group, 23.7 (95% CI 20.9–26.5; *p* = 0.001 compared to full treatment) days for the withholding group, and 28.7 (95% CI 28.3–29.2) days for the full treatment group (Supporting Information, pages 9–10, 13). Of the patients undergoing withdrawal of active treatment, only 1.6% (*n* = 2) survived for more than five days after the TLD, corresponding to a difference in 30‐day mean restricted survival time of 20.1 (95% CI, 16.9–23.4, *p* < 0.001) days after the TLD compared with patients subjected to withholding (Figure 4; Supporting Information, Page 13).

A Cox proportional‐hazards analysis of 30‐day mortality after admission and including all three treatment levels with full treatment as the reference yielded HRs of 70 for withdrawal (95% CI, 38.5–127.2; *p* < 0.001) and 3.6 (95% CI, 1.3–10.4; *p* = 0.02) for withholding (Supporting Information, Page 8), respectively. For both withholding and withdrawal, we found an HR of 1.2 (95% CI, 1.1–1.3; *p* = 0.005) for the Charlson Comorbidity Index. A model including only the two TLD groups with withholding as a reference yielded an HR of 13 for withdrawal (95% CI, 5–31.9; *p* < 0.001) (Supporting Information, Page 12).

**FIGURE 5:**
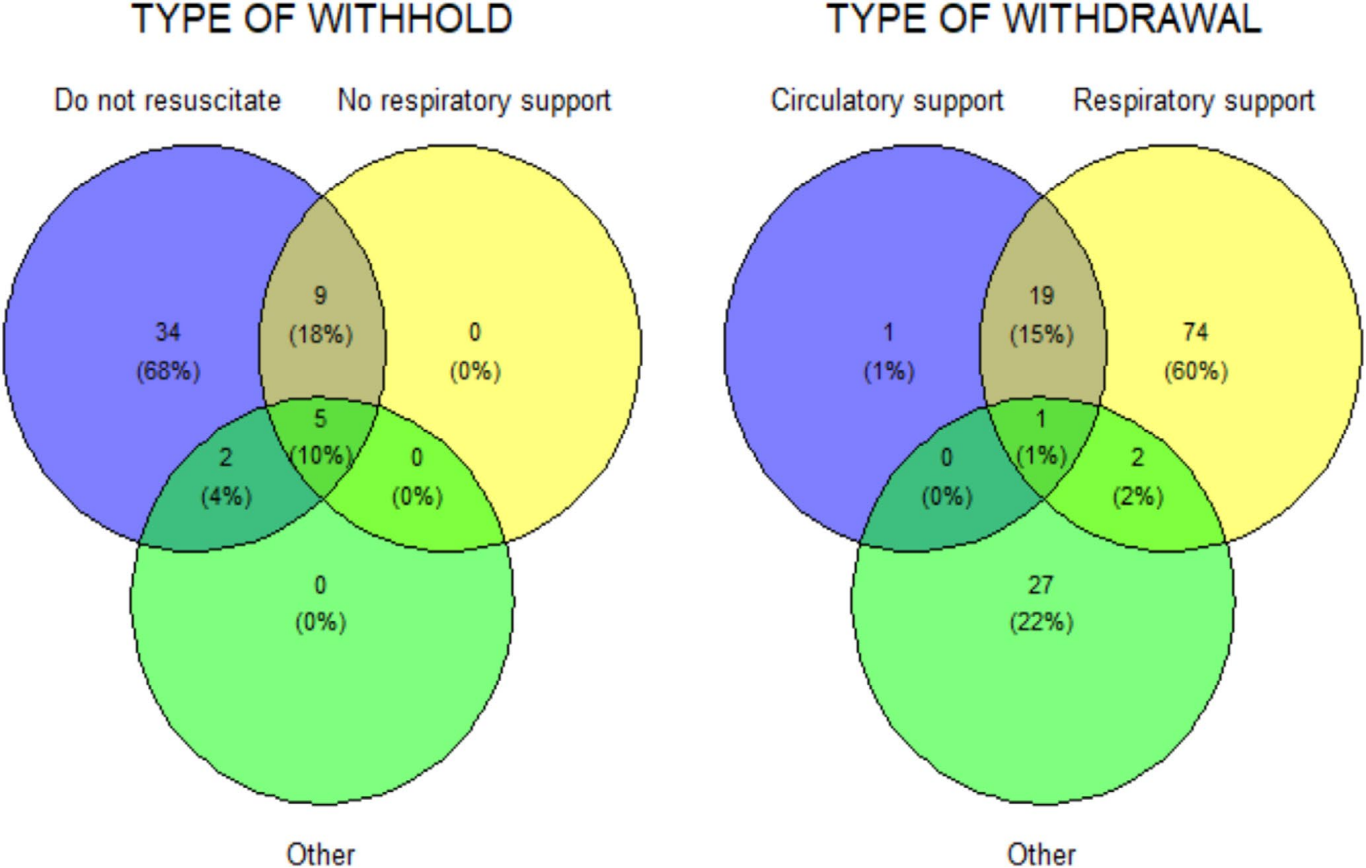
Types of TLD. Withholding: ‘Other’ contains ‘no pacemaker’, ‘no further escalation of treatment already initiated’, ‘no dialysis’, ‘no further operations like hemicraniectomy or endovascular thrombectomy’. Withdrawal: ‘Other’ contains ‘no dialysis’, ‘no antibiotic treatment’, ‘no further treatment in case of increasing intracranial pressure’, ‘no treatment of coagulopathy’, ‘no treatment of gastrointestinal bleeding’ and change of therapy with a focus on survival to that of organ preservation in patients who went on to become organ donors (*n* = 30).

An overview of specific treatments addressed in decisions to withhold and withdraw is shown in Figure 5. All patients in the withholding group had a ‘do not resuscitate’ (DNR) order, of whom 68% had a DNR order as the sole treatment limitation. Withdrawal of respiratory support was the main decision for patients subjected to withdrawal.

## 4 Discussion

In this retrospective study of patients admitted to the neuro‐ICU, a TLD was recorded for 25% of whom one‐third were subjected to withholding and two‐thirds to withdrawal of treatment. As stated in the introduction, we initially formulated a zero hypothesis that mortality rates for withholding and withdrawal would be comparable. Thus, different authors had different a priori assumptions and reasoning regarding the relative effect of withholding versus withdrawal on mortality. The argument supporting a higher mortality after withdrawal compared to withholding stated withdrawal would occur in patients who all had shown a need for organ support, whether a fraction of those in the withholding group might never develop such a need. Conversely, the argument supporting a higher mortality after withholding compared to withdrawal reasoned that withholding often occurs early in the clinical course when prognosis remains uncertain, whereas withdrawal typically follows a period of aggressive treatment during which improvement could have occurred (but did not).

In 2003, Azoulay and colleagues reported that of 1385 mixed‐ICU patients who were discharged alive from seven ICUs in or near Paris, France, after a minimum of 48 h of intensive care, only 80 (5.7%) were subjected to ICU‐sanctioned decisions to withhold or withdraw life‐sustaining care, of whom 47 died in hospital [9]. In fact, a TLD was associated with an odds ratio of 9.64 of dying after ICU discharge, rendering it the single most important predictor of post‐ICU mortality. Our study data are in agreement with this notion of a strong impact of TLDs on early mortality. Furthermore, in 2019, the European prospective observational multi‐centre ETHICUS‐2 study reported that the fraction of mixed‐ICU patients subjected to a TLD increased from approximately 9% in 1999–2000 to approximately 12% in 2015–2016 [10]. Thus, the use of TLDs has increased over time; however, at 25%, the prevalence of TLDs in our patients was several times as high as that in the mixed cohorts described above. As our patients were comparatively younger than these cohorts, age does not explain this difference. Rather, we speculate that the frequent use of TLDs was disease‐specific; thus, in ETHICUS‐2, compared to other types of critical illness, acute neurological disease was associated with the highest odds ratio of TLDs [11].

Primary neurological injury was a reason for TLD in almost all patients subjected to withdrawal of treatment; treatment was withheld because of the primary injury in 68%, while comorbidities were cited as a reason in 46%. Patients subjected to withholding were older and had more comorbidities than the two other groups, while those undergoing withdrawal had higher disease severity and lower initial levels of consciousness. At 30 days after admission, almost all patients subjected to withdrawal of therapy had died, compared to one‐third of patients subjected to withholding and less than one in ten patients receiving full treatment. Withdrawal usually included discontinuation of ventilation, which together with other means of support is a common life supporting measure; hence, death cannot be unexpected and poor prognosis after withdrawal of life support is a self‐fulfilling prophecy [4]. For this reason, withdrawal should not be undertaken unless the prognostic uncertainty has been minimised. Faiver et al. emphasise limitations of prognostic models and risk of premature decisions ‘before at least 72 h [4]. In the present study, withdrawal was undertaken after due aggressive treatment, and it appears that even severely injured patients received aggressive treatment until prolonged observation led to a re‐evaluation to discontinuation of care.

In contrast, the practice of withholding raised more questions as aggressive care would not necessarily have been ‘futile’. These patients were older and had more comorbidities, yet two thirds of the patients survived 30 days despite the decision to withhold aggressive care. Whether these patients would have benefitted from more aggressive care and whether survival would have been even greater if aggressive care had been initiated is an open question. Longer follow‐up and detailed analyses of neurological outcomes are needed, although a provisional observation could be that early aggressive management could be motivated unless the initial injury, in conjunction with the patient's comorbidities (if any), can be safely assessed to be incompatible with reasonable neurological recovery.

The benchmark of TLDs is difficult, as culture and practice vary and treatment limitations can be more or less explicit and traceable [12]. Thus, the prevalence of TLD varies widely in the literature. In CENTER‐TBI—a recent multi‐centre, European study of traumatic brain injury—the occurrence of TLD among TBI patients who died during their stay at the ICU varied from 0% to 96% between hospitals, with an average prevalence of 10% [13]. Besides local differences in case mix and therapeutic activity for patients at high risk of unfavourable outcomes, this finding is likely also due to different traditions of when and how TLD are recorded, once they have been made during the course of illness. The underlying ethical deliberation is existential, as it reflects upon how the value of life is perceived: if the value of life is intrinsic, or if life has value only as long as it is a means to experience and interact with others. Most care providers in European countries would not consider initiating or continuing treatment to be reasonable unless a patient is likely to regain consciousness and some degree of independence [12]. Another ethical issue is prognostication in the face of uncertainty. Prognostic algorithms of outcomes cannot readily inform individual prediction and frequently fail external validation [14, 15, 16]; it is also possible that medical decision‐making can be influenced by irrelevant confounders and prejudice [17].

In a large American, register‐based study based on reporting to the Nationwide Inpatient Sample, withdrawal of care was done in 3.4% of all patients with SAH [18]. This is substantially lower than our findings where withdrawal occurred in 12% of SAH patients. This study may not be directly comparable to our study, as it was conducted in 2001–2010 and was based on reporting to an external registry (using International Classification of Diseases classification), while the present study was based on medical records where explicit, traceable data were necessary to legally document clinical decision‐making. Moreover, culturally, American culture is more inclined to consider life as an intrinsic value, and American physicians might therefore be less inclined to follow an explicit TLD [19]. Even so, in some agreement with our findings, patients subjected to withdrawal were older, had more comorbidities and in‐hospital complications and had a higher prevalence of severe disability; their in‐hospital mortality was 80% compared to 21% in those receiving full treatment.

The prevalence of TLD also appears to vary in ICH patients; in one California‐based study of ICH patients admitted via the emergency department, the rate of do‐not‐resuscitate decisions within 24 h after admission varied from 0% to 70% between hospitals, for an average of 25% [20]; this variability persisted after adjustment for case mix and was a strong driver of mortality (95% CI, 2.07–5.19; *p* < 0.001); other risk factors were mechanical ventilation (95% CI, 12.54–16.46; *p* < 0.001) and age (95% CI, 1.18–1.31; *p* < 0.001). Conversely, in an Australian study of ICH patients who were included in the INTERACT‐2 study, only 4% of patients underwent withdrawal of active treatment [21]; this decision was associated with higher age, greater disease severity and randomisation to a lower rather than a higher blood pressure. In comparison, the rate of TLD in ICH patients in the present study was nearly 34%. This likely reflects key differences in study populations, as INTERACT‐2 excluded patients with severe ICH (GCS < 5 or haematoma volume ≥ 60 cm^3^), thereby enrolling a less critically ill cohort not typically managed in a neurointensive setting.

It is important to note that all the cited studies on ICH and SAH included mixed patient populations, with both ICU and non‐ICU patients. In contrast, our study focused exclusively on neurointensive care patients. This difference limits direct comparability, as non‐ICU patients in the referenced studies may have been either too well to require intensive care or too severely affected to benefit from it. Consequently, TLDs in our cohort reflect decision‐making in the context of intensive care, which likely influences both the frequency and nature of such decisions.

Not surprisingly, cessation of mechanical ventilation was the most frequent action in patients subjected to withdrawal. Thus, mechanical ventilation is both a marker of illness severity and a life‐sustaining intervention. Withdrawal of respiratory support in ventilator‐dependent patients, usually by extubation of intubated patients [22], would be expected to lead to rapid respiratory failure and eventually death in most patients. This also speaks to the ‘self‐fulfilling prophecy’ associated with withdrawal of mechanical ventilation in patients with a perceived poor prognosis [3]. In contrast, withholding does not entail extubation and therefore has less dramatic consequences.

Studies of TLDs do not always distinguish between withholding and withdrawal of treatment [13, 18, 21, 23]. For example, in their retrospective study of a large cohort of mechanically ventilated, brain‐injured patients [24], Nesseler et al. did not distinguish between withholding and withdrawal in their data extraction from a large cohort of mechanically ventilated, brain‐injured patients [24], but reported that older age and disease severity were associated with a decision withhold or withdraw therapy within three months after the injury. In contrast to that study, Robertsen et al. distinguished between several different types of TLD in TBI patients, such as ‘do not resuscitate’, ‘withholding of surgery’ and ‘no escalation of treatment’ for withholding, and ‘withdrawal of organ support’ for withdrawal [25]. The in‐hospital mortality was 73% and 1% for patients with and without any TLD, respectively. Conversely, the study by Hemphill et al. focused primarily on ‘do not resuscitate’ orders in patients with intracerebral haemorrhage [20] and found higher in‐hospital mortality in patients who underwent this decision early during their stay. Our data suggest that both withholding and withdrawal are associated with different injury severity and outcomes. Moreover, from a perspective of ethical agency, different ethical systems may have different implications. From a consequentialist perspective, withholding and discontinuing would be equal, while other systems may view discontinuation as an active measure to limit a patient's survival. In the latter systems, if active discontinuation is difficult or impossible, for example by law, responsible physicians may be overly reluctant to initiate aggressive treatment. Paradoxically, the more cynical consequentialist attitude may thus allow more patients with unclear prognoses to be offered ‘a chance’ to respond positively to aggressive therapy.

In a similar population, Diringer et al. reported that a low admission GCS, high APACHE II and age, but also diagnoses such as SAH or another type of stroke and not having undergone surgery, were independent risk factors for withdrawal of mechanical ventilation [26]. In the more recent CENTER‐TBI investigation of patients with TBI, a high injury severity score was also associated with withdrawal of life‐sustaining measures [13]. Thus, despite the widely acknowledged challenge regarding early prognostication in patients with severe acute brain injury [8, 27, 28], more severe injury is generally associated with a higher risk of imposing a TLD.

In our cohort, age was not reported as a common reason for a TLD. Several previous studies have reported age as an associated factor in TLDs [20, 24, 26, 28]. Whether our observation indicates an increasing focus over time on disability and frailty [29] for individual prognostication, rather than more general risk factors such as age, remains to be studied. It is of interest to analyse whether treatment may have been withheld based on preconceived expectations of a bad prognosis due to age, as withholding treatment was associated with age, although survival in the group of ‘withheld treatment’ was unexpectedly high. A possible explanation is that very old or frail patients, or those with severe comorbidities, may have been excluded from intensive care already in the emergency department, thereby attenuating the influence of age on later TLD decisions within the neuro‐ICU.

Comorbidities were reported as an additional reason for withholding and, to a lesser extent, for withdrawal. Interestingly, Gambhir et al. observed that patients who did survive withdrawal of life‐sustaining measures were older and had more comorbidities than those who did not survive [6]. However, the definition of withdrawal in that study seemed to entail both withdrawal and withholding as defined in our study. Thus, the data are not immediately comparable.

The median duration from neuro‐ICU admission to a TLD was 28 and 36 h for the withholding and withdrawal group, respectively. The Neurocritical Care Society has recommended that withdrawal of life‐sustaining measures should be postponed until 72 h after the injury in patients with devastating brain injury to avoid variability in outcome [1]. However, there is great variability in the timing of TLDs and prognostication regarding patients with TBI and other brain injuries [8, 13, 28, 30], probably reflecting the difficult choice between continuing active treatment with the associated risk of survival with an unfavourable outcome and limiting treatment with the associated risk of a self‐fulfilling prophecy [8, 31, 32]. Overestimation of the risk of death in neuro‐ICU patients has been associated with a greater probability of a TLD and decreasing intensity of treatment [8]. However, the anticipated patient recovery is also a critical part of the decision process [23]. Studies have shown that physicians within the same department may have different perspectives in assessing clinical cases and cite different factors which they consider of importance in prognostication [30, 33]. This can lead to an undesirable variability in treatment. Such variability probably reflects that ethical and medical issues of discontinuation or withholding care are not transparently addressed in everyday decision‐making. We have already argued that prognostication is imprecise. Moreover, different ethical tools may yield different practical conclusions. In medicine, the four principles of Childress and Beauchamp are common tools [34]. In this context, we have argued that ‘autonomy’, ‘benefit of the patient’ and ‘not harming the patients’ are of little use, as patient's perspectives remain unclear in terms desirable outcomes and suffering; these qualities are subjective and cannot readily be assessed by medical professionals. In contrast, the principle of justice is relevant; it suggests that treatment should be generalisable to all patients. It may not be possible for all potential patients to first use extensive resources for very aggressive treatment to prolong a patient's life if the resulting condition requires additional extensive resources to sustain life. From an ethical perspective, the practice to aggressively treat patients until prognosis becomes clear seems to be preferable to withholding treatment without strong evidence of futility.

### 4.1 Strengths and Limitations

A major strength of this study is the completeness of data, as well as our ability to distinguish between withholding and withdrawal of life‐sustaining measures based on the case notes.

Based on the notion that the decision processes for treatment levels depend on the severity of acute neurological injury rather than the underlying type of injury, we elected to describe a cohort including different disease categories. This increased the sample size, which is a strength; however, some may view the mixed cohort as a limitation because different pathophysiological processes and structural injuries are pooled. The width of the CI reflects the small sample size in the subgroups and the considerable difference in event rates, particularly the high mortality in the withdrawal group. The findings should therefore be interpreted with caution, and future larger studies are warranted to confirm these estimates.

This was a single‐centre study, which may reduce external validity; different hospitals may nurture different cultures for deciding the level of treatment and favour different algorithms for TLDs. The retrospective nature of the study means we were restricted in our data extraction by the existing entries by clinicians; we cannot exclude the possibility that TLDs were made for some patients without an accompanying entry in the case notes. The study was exploratory, so no correction for multiple testing was applied in the statistical analyses; accordingly, results should be considered hypothesis‐generating rather than definitive.

To minimise dropout and censoring of the most recently admitted patients, we analysed 30‐day mortality rather than applying a longer time span. However, it could also be interesting to look at functional outcome and mortality in a longer timeframe [23]. We did not include data on pre‐admission frailty or daily functioning, as this information was not consistently available in the medical records. While the Charlson Index captures comorbidity, functional status before admission may be a more relevant factor in TLD decisions and should be considered in future studies.

Another limitation is that we used the standard Charlson Comorbidity Index, which includes age as a component. Since patients in the withholding group were older, part of the observed difference in comorbidity burden may reflect age rather than underlying disease alone. A modified Charlson Comorbidity Index excluding age could have provided additional insight.

Part of the study was conducted during the COVID pandemic, where significant strain on the ICUs and their staff may have occurred, and where subtle changes in decision‐making processes may have affected the use of TLDs. However, Denmark was relatively less affected than most other European countries, the Neuro‐ICU received a very limited number of COVID patients, and patients with a need for highly specialised treatment were accepted even at the height of the pandemic without additional restrictions; furthermore, the available data suggest that the mortality of non‐COVID patients admitted to Danish ICUs was unchanged in 2020 compared to 2019 [35].

Our registration of mechanical ventilation was done by checking the absence or presence of a specific data entry, which may have been forgotten by the clinicians responsible for this entry, in particular for shorter ICU stays. Therefore, we may have underestimated the proportion of mechanically ventilated patients, in particular in the full‐treatment and the withdrawal group, because of the generally shorter length of stay in these groups. The time of TLD was extracted from the patient files as the time of the note, which might include a slight delay between decision and registration. Patients who went on to become donors were included in the withdrawal group instead of excluding this group altogether. This was done based on the argument that if they had not become organ donors, treatment would have been withdrawn after clinical brain death occurred. Thus, excluding them would have skewed the data of the withdrawal group.

Finally, as the study was considered to be hypothesis‐generating and exploratory, we chose not to correct for multiple comparisons, which increases the risk of type 1 errors. Accordingly, the results of the present study should be tested in the future using more rigorous designs.

## 5 Conclusion

In this study of TLDs in the neuro‐ICU, patients subjected to withholding and withdrawal of life‐sustaining measures, respectively, had different demographics and survival. Whereas the primary neurological injury was the single dominant reason for deciding to withdraw life‐sustaining measures, both the primary neurological injury and, to a lesser extent, non‐neurological injury were mentioned as reasons to withhold life‐sustaining measures. Comorbidities also played a role in the decision to withdraw and, in particular, to withhold life‐sustaining measures. Cardiac arrest in conjunction with the ictus or injury was an important additional reason to withdraw life‐sustaining measures.

We suggest distinguishing between withholding and withdrawal of active treatment in future studies of treatment‐limiting decisions.

## Author Contributions

K.M., M.H.O., A.‐S.W.F. and I.C.A. conceived the study. I.C.A., M.H.O. and H.R.J. participated in data extraction. All authors commented on the manuscript, made significant adjustments and approved the final version of the manuscript.

## Funding

The authors have nothing to report.

## Conflicts of Interest

The authors declare no conflicts of interest.

## Supporting information


**Data S1:** Supplementary figures and analyses are provided in the Supporting Information.

## Data Availability

The data that support the findings of this study are available on request from the corresponding author. The data are not publicly available due to privacy or ethical restrictions.
