# Supplementary material for: Treatment‐Limiting Decisions in Neurointensive Care: Withholding or Withdrawal of Life‐Sustaining Measures
Source: Acta Anaesthesiol Scand. 2026 Feb 22;70(3):e70202. doi: 10.1111/aas.70202 (PMC12925652; doi:10.1111/aas.70202)

Supplemental Material for:

Treatment-limiting decisions in neurointensive care: withholding or withdrawal of life-sustaining measures

# **Contents** Table 1: Demographic data and patient characteristics page 2 Table 2: Reasons for treatment-limiting decisions page 5

# Figure 1 - Survival from Admission; Schoenfeld individual test page 7

# Hazard Ratio; full treatment, withholding and withdrawal of treatment page 8

# 30-day mortality after admission to the neuro-ICU (Kaplan-Meier plot) page 8 Restricted Mean Survival Time: full vs withholding of treatment page 9 Restricted Mean Survival Time: withdrawal of treatment vs full treatment page 10 Figure 2 - Survival from TLD, Shoenfeld Individual Test page 11 Hazard Ratio, withholding and withdrawal of treatment page 12 30-day mortality after a treatment-limiting decision (Kaplan-Meier plot) page 12 Restricted Mean Survival Time: withholding vs withdrawal of treatment page 13 Venn diagram; types of TLD page 14

# Table 1: Demographic data and patient characteristics

## Stratified by group
## Full
## n 522
## age (median [IQR]) 56.50 [46.00, 66.00]
## sex = Male (%) 281 (53.8)
## charlson_sum (median [IQR]) 2.00 [1.00, 4.00]
## diagnose (%)
## aSAH 75 (14.4)
## Other (cerebrovascular/tumour/SCI) 151 (28.9)
## Other (neurological/neuroinfection/neuroinflammation) 89 (17.0)
## sICH 129 (24.7)
## TBI 78 (14.9)
## tdl_first_gcs (median [IQR]) 14.00 [9.00, 15.00]
## abi_apacheii (median [IQR]) 18.00 [13.00, 23.00]
## mechan_vent = Yes (%) 278 (53.3)
## ictus_to_adm (median [IQR]) 2.00 [0.80, 15.43]
## adm_to_nicu (median [IQR]) 3.34 [0.53, 17.77]
## los (median [IQR]) 3.85 [1.45, 10.53]
## mors_30_day = Yes (%) 51 ( 9.8)
## Stratified by group
## Withdrawal
## n 124
## age (median [IQR]) 60.00 [51.75, 67.00]
## sex = Male (%) 72 (58.1)
## charlson_sum (median [IQR]) 3.00 [2.00, 4.00]
## diagnose (%)
## aSAH 19 (15.3)
## Other (cerebrovascular/tumour/SCI) 29 (23.4)
## Other (neurological/neuroinfection/neuroinflammation) 23 (18.5)
## sICH 41 (33.1)
## TBI 12 ( 9.7)
## tdl_first_gcs (median [IQR]) 6.00 [3.00, 13.00]
## abi_apacheii (median [IQR]) 23.00 [20.00, 26.00]
## mechan_vent = Yes (%) 107 (86.3)
## ictus_to_adm (median [IQR]) 1.24 [0.68, 4.45]
## adm_to_nicu (median [IQR]) 1.51 [0.15, 6.06]
## los (median [IQR]) 2.24 [1.30, 4.94]
## mors_30_day = Yes (%) 121 (97.6)
## Stratified by group
## Withhold
## n 50
## age (median [IQR]) 66.00 [48.00, 74.75]
## sex = Male (%) 24 (48.0)
## charlson_sum (median [IQR]) 4.00 [3.00, 6.00]
## diagnose (%)
## aSAH 6 (12.0)
## Other (cerebrovascular/tumour/SCI) 15 (30.0)
## Other (neurological/neuroinfection/neuroinflammation) 6 (12.0)
## sICH 18 (36.0)
## TBI 5 (10.0)
## tdl_first_gcs (median [IQR]) 12.00 [7.00, 15.00]
## abi_apacheii (median [IQR]) 20.00 [16.25, 24.00]
## mechan_vent = Yes (%) 33 (66.0)
## ictus_to_adm (median [IQR]) 1.85 [0.98, 16.69]
## adm_to_nicu (median [IQR]) 2.84 [0.66, 31.72]
## los (median [IQR]) 9.07 [3.23, 17.66]
## mors_30_day = Yes (%) 16 (32.0)

|  | Full | Withhold | Withdrawal |
| --- | --- | --- | --- |
| n | 522 | 50 | 124 |
| age (median [IQR]) | 56.50 [46.00, 66.00] | 66.00 [48.00, 74.75] | 60.00 [51.75, 67.00] |
| sex = Male (%) | 281 (53.8) | 24 (48.0) | 72 (58.1) |
| charlson_sum (median [IQR]) | 2.00 [1.00, 4.00] | 4.00 [3.00, 6.00] | 3.00 [2.00, 4.00] |
| diagnose (%) |  |  |  |
| aSAH | 75 (14.4) | 6 (12.0) | 19 (15.3) |
| Other (cerebrovascular/tumour/SCI) | 151 (28.9) | 15 (30.0) | 29 (23.4) |
| Other (neurological/neuroinfection/neuroinflammation) | 89 (17.0) | 6 (12.0) | 23 (18.5) |
| sICH | 129 (24.7) | 18 (36.0) | 41 (33.1) |
| TBI | 78 (14.9) | 5 (10.0) | 12 ( 9.7) |
| tdl_first_gcs (median [IQR]) | 14.00 [9.00, 15.00] | 12.00 [7.00, 15.00] | 6.00 [3.00, 13.00] |
| abi_apacheii (median [IQR]) | 18.00 [13.00, 23.00] | 20.00 [16.25, 24.00] | 23.00 [20.00, 26.00] |
| mechan_vent = Yes (%) | 278 (53.3) | 33 (66.0) | 107 (86.3) |
| ictus_to_adm (median [IQR]) | 2.00 [0.80, 15.43] | 1.85 [0.98, 16.69] | 1.24 [0.68, 4.45] |
| adm_to_nicu (median [IQR]) | 3.34 [0.53, 17.77] | 2.84 [0.66, 31.72] | 1.51 [0.15, 6.06] |
| los (median [IQR]) | 3.85 [1.45, 10.53] | 9.07 [3.23, 17.66] | 2.24 [1.30, 4.94] |
| mors_30_day = Yes (%) | 51 ( 9.8) | 16 (32.0) | 121 (97.6) |

## patient admission: 2019-07-03 01:34:00 2022-02-16 23:42:00

## Age | Wilcox Full vs. Withhold - p.val: 0.001111791

## Age | Wilcox Full vs. Withdraw - p.val: 0.05466961

## Age | Wilcox Withhold vs. Withdraw - p.val: 0.03057567

## GCS | Wilcox Full vs. Withhold - p.val: 0.08991059

## GCS | Wilcox Full vs. Withdraw - p.val: 6.367764e-17

## GCS | Wilcox Withhold vs. Withdraw - p.val: 0.0001063312

## APACHE-II | Wilcox Full vs. Withhold - p.val: 0.06005377

## APACHE-II | Wilcox Full vs. Withdraw - p.val: 7.113416e-14

## APACHE-II | Wilcox Withhold vs. Withdraw - p.val: 0.001670966

## Charlson | Wilcox Full vs. Withhold - p.val: 1.197308e-06

## Charlson | Wilcox Full vs. Withdraw - p.val: 0.005021836

## Charlson | Wilcox Withhold vs. Withdraw - p.val: 0.00365753

# Table 2: Reasons for treatment-limiting decisions

## Stratified by group
## Withdrawal
## n 124
## nicu_to_tld (median [IQR]) 35.65 [15.09, 98.84]
## TLD_reason (%)
## Neurological damage (primary) 119 (96.0)
## Neurological damage (secondary) 1 ( 0.8)
## Non-neurological damage 3 ( 2.4)
## Unknown 1 ( 0.8)
## TLD_additional_reason_age = Yes (%) 1 ( 0.8)
## TLD_additional_reason_comorbidities = Yes (%) 27 (21.8)
## TLD_additional_reason_primary_OHCA = Yes (%) 20 (16.1)
## Stratified by group
## Withhold
## n 50
## nicu_to_tld (median [IQR]) 28.15 [15.23, 218.33]
## TLD_reason (%)
## Neurological damage (primary) 34 (68.0)
## Neurological damage (secondary) 0 ( 0.0)
## Non-neurological damage 8 (16.0)
## Unknown 8 (16.0)
## TLD_additional_reason_age = Yes (%) 1 ( 2.0)
## TLD_additional_reason_comorbidities = Yes (%) 23 (46.0)
## TLD_additional_reason_primary_OHCA = Yes (%) 2 ( 4.0)

|  | Withhold | Withdrawal |
| --- | --- | --- |
| n | 50 | 124 |
| nicu_to_tld (median [IQR]) | 28.15 [15.23, 218.33] | 35.65 [15.09, 98.84] |
| TLD_reason (%) |  |  |
| Neurological damage (primary) | 34 (68.0) | 119 (96.0) |
| Neurological damage (secondary) | 0 ( 0.0) | 1 ( 0.8) |
| Non-neurological damage | 8 (16.0) | 3 ( 2.4) |
| Unknown | 8 (16.0) | 1 ( 0.8) |
| TLD_additional_reason_age = Yes (%) | 1 ( 2.0) | 1 ( 0.8) |
| TLD_additional_reason_comorbidities = Yes (%) | 23 (46.0) | 27 (21.8) |
| TLD_additional_reason_primary_OHCA = Yes (%) | 2 ( 4.0) | 20 (16.1) |

# Figure 1 - Survival from Admission; Schoenfeld individual test
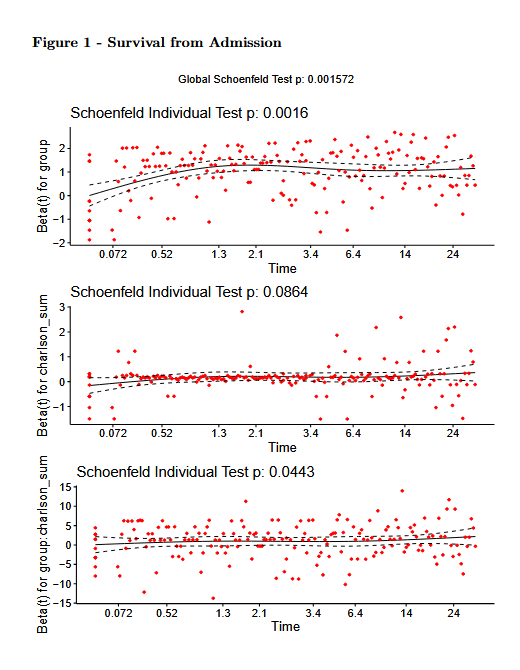


# Hazard Ratio; full treatment, withholding and withdrawal of treatment

#
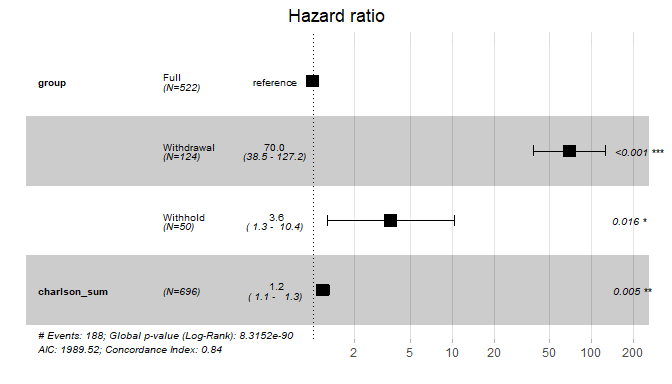


# 30-day mortality after admission to the neuro-ICU (Kaplan-Meier plot)
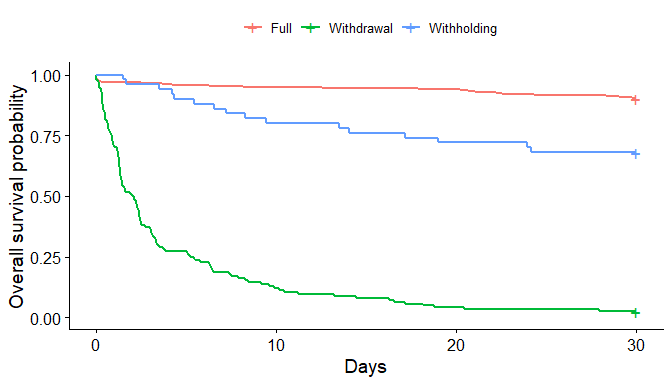


## Restricted Mean Survival Time: full vs withholding of treatment

## FULL = 0, WITHHOLD = 1


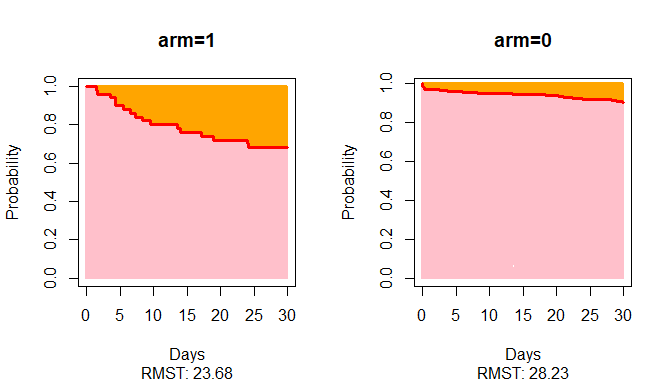


##
## The truncation time, tau, was not specified. Thus, the default tau 30 is used.
##
## Restricted Mean Survival Time (RMST) by arm
## Est. se lower .95 upper .95
## RMST (arm=1) 23.682 1.427 20.886 26.478
## RMST (arm=0) 28.229 0.280 27.680 28.779
##
##
## Restricted Mean Time Lost (RMTL) by arm
## Est. se lower .95 upper .95
## RMTL (arm=1) 6.318 1.427 3.522 9.114
## RMTL (arm=0) 1.771 0.280 1.221 2.320
##
##
## Between-group contrast
## Est. lower .95 upper .95 p
## RMST (arm=1)-(arm=0) -4.548 -7.397 -1.698 0.002
## RMST (arm=1)/(arm=0) 0.839 0.744 0.946 0.004
## RMTL (arm=1)/(arm=0) 3.569 2.078 6.127 0.000

## FULL = 0, WITHDRAW = 1

## Restricted Mean Survival Time: withdrawal of treatment vs full treatment


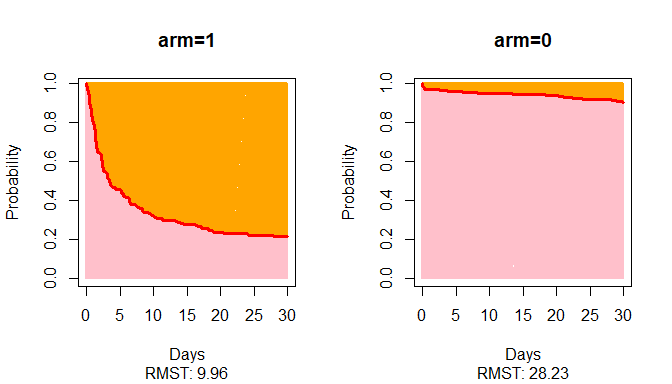


##
## The truncation time, tau, was not specified. Thus, the default tau 30 is used.
##
## Restricted Mean Survival Time (RMST) by arm
## Est. se lower .95 upper .95
## RMST (arm=1) 9.959 0.876 8.241 11.677
## RMST (arm=0) 28.229 0.280 27.680 28.779
##
##
## Restricted Mean Time Lost (RMTL) by arm
## Est. se lower .95 upper .95
## RMTL (arm=1) 20.041 0.876 18.323 21.759
## RMTL (arm=0) 1.771 0.280 1.221 2.320
##
##
## Between-group contrast
## Est. lower .95 upper .95 p
## RMST (arm=1)-(arm=0) -18.270 -20.074 -16.467 0
## RMST (arm=1)/(arm=0) 0.353 0.297 0.420 0
## RMTL (arm=1)/(arm=0) 11.319 8.202 15.620 0

# Figure 2 - Survival from TLD, Shoenfeld Individual Test
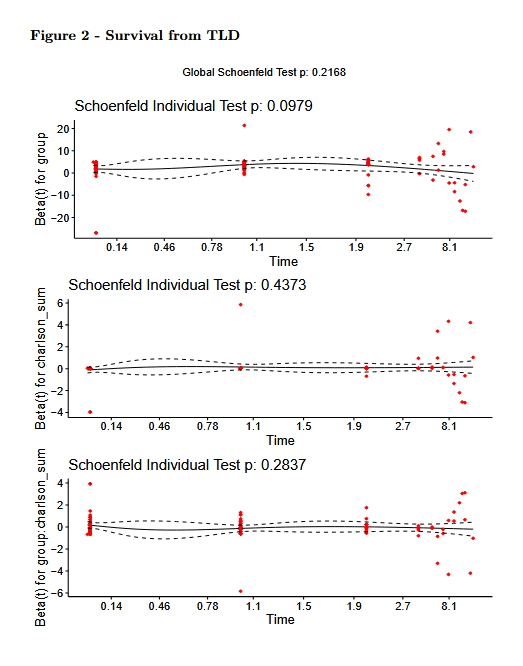


# Hazard Ratio, withholding and withdrawal of treatment

#
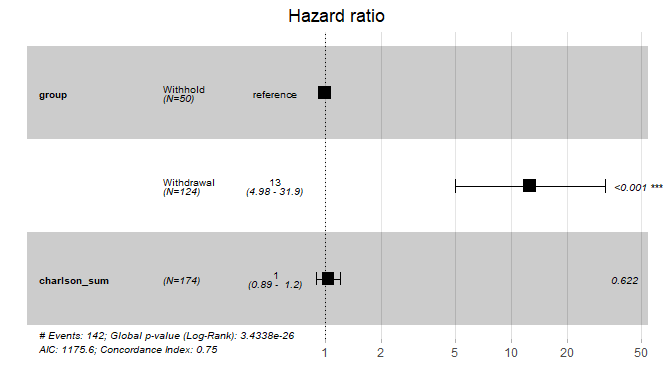


# 30-day mortality after a treatment-limiting decision (Kaplan-Meier plot)
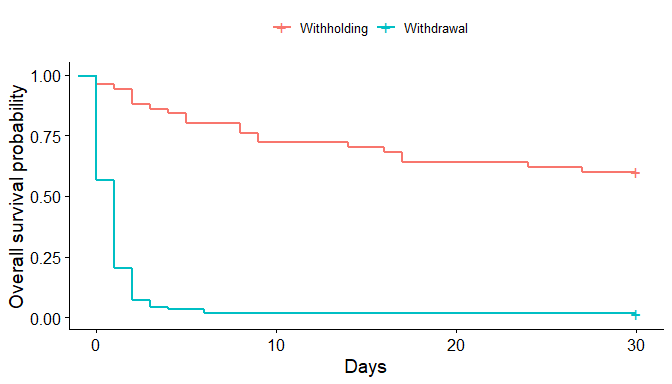


## [1] "Survivors more than 5 days after TLD:"

## [1] 0.01612903

## Restricted Mean Survival Time: withholding vs withdrawal of treatment

## WITHHOLD = 0, WITHDRAW = 1


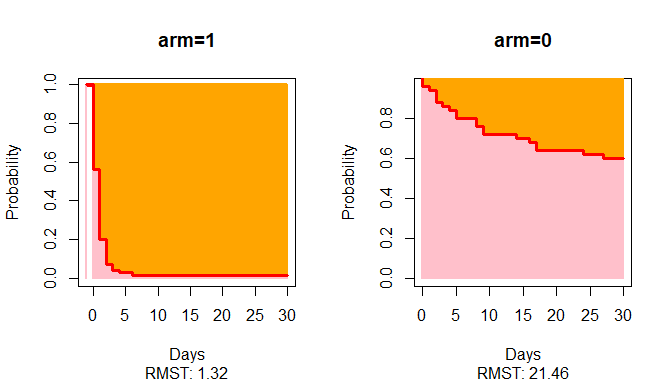


##
## The truncation time, tau, was not specified. Thus, the default tau 30 is used.
##
## Restricted Mean Survival Time (RMST) by arm
## Est. se lower .95 upper .95
## RMST (arm=1) 1.323 0.344 0.649 1.996
## RMST (arm=0) 21.460 1.635 18.255 24.665
##
##
## Restricted Mean Time Lost (RMTL) by arm
## Est. se lower .95 upper .95
## RMTL (arm=1) 28.677 0.344 28.004 29.351
## RMTL (arm=0) 8.540 1.635 5.335 11.745
##
##
## Between-group contrast
## Est. lower .95 upper .95 p
## RMST (arm=1)-(arm=0) -20.137 -23.413 -16.862 0
## RMST (arm=1)/(arm=0) 0.062 0.036 0.105 0
## RMTL (arm=1)/(arm=0) 3.358 2.305 4.891 0

Venn diagram; types of TLD


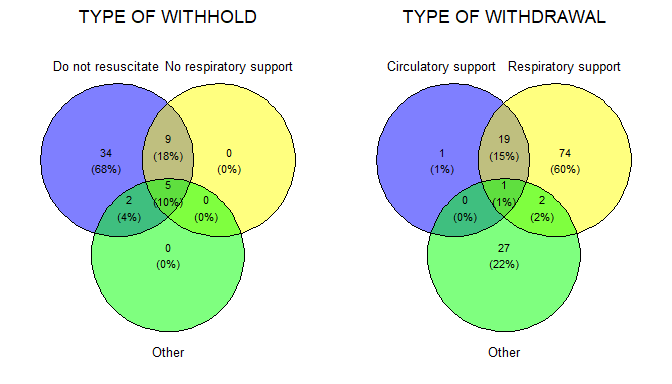

Supplement: Supplementary file 1 — Data S1: Supplementary figures and analyses are provided in the Supporting Information. [file AAS-70-0-s001.docx]
